# Computational Study of In-Plane Phonon Transport in Si Thin Films

**DOI:** 10.1038/srep06399

**Published:** 2014-09-17

**Authors:** Xinjiang Wang, Baoling Huang

**Affiliations:** 1 Department of Mechanical and Aerospace Engineering, The Hong Kong University of Science and Technology, Clear Water Bay, Kowloon, Hong Kong; 2 The Hong Kong University of Science and Technology Shenzhen Research Institute, Shenzhen, 518057, China

## Abstract

We have systematically investigated the in-plane thermal transport in Si thin films using an approach based on the first-principles calculations and lattice dynamics. The effects of phonon mode depletion induced by the phonon confinement and the corresponding variation in interphonon scattering, which may be important for the thermal conductivities of ultra-thin films but are often neglected in precedent studies, are considered in this study. The in-plane thermal conductivities of Si thin films with different thicknesses have been predicted over a temperature range from 80 K to 800 K and excellent agreements with experimental results are found. The validities of adopting the bulk phonon properties and gray approximation of surface specularity in thin film studies have been clarified. It is found that in ultra-thin films, while the phonon depletion will reduce the thermal conductivity of Si thin films, its effect is largely offset by the reduction in the interphonon scattering rate. The contributions of different phonon modes to the thermal transport and isotope effects in Si films with different thicknesses under various temperatures are also analyzed.

Single crystalline silicon thin films have been widely used in microfabricated sensors, actuators, and transistors^1^,^2^. The efforts in reducing the power consumption and miniaturizing microelectronic devices entail even thinner silicon thin films. Ultra-thin Si films with a thickness less than 12 nm have been fabricated and applied to Silicon-On-Insulator (SOI) transistors^3^ to lower drive voltages. However, experiments show that attenuating the film thickness below submicron can significantly suppress the in-plane thermal transport, resulting in a thermal conductivity several factors or even an order of magnitude lower than that of the corresponding bulk material^4^,^5^,^6^. The reduction of thermal conductivity in Si thin films can deteriorate the heat dissipation process in electronic devices such as SOI-based microprocessors^7^, light-emitting diode (LED) and differential scanning nanocalorimeters^8^, where good lateral heat conduction is crucial for efficient thermal management, fast thermal response and good sensitivity. On the other hand, this size effect may serve as a promising solution to enhance the figure of merit when applied to thermoelectric devices^9^. Understanding and predicting spectral thermal transport in thin films is essential to further the advance of the nanoelectronics and thermoelectrics. However, currently the measurements of in-plane thermal conductivity of Si thin films haven't forged deeper into the sub-20 nm regime^5^, calling for theoretical efforts to investigate the phonon transport in such ultra-thin films.

With the attenuation of film thickness, phonon scattering at the film boundaries becomes relatively more important in suppressing phonon mean free path (MFP). This has been widely accepted as the main reason for the reduction of thermal conductivity in nanostructured materials^10^,^11^. Early-stage investigations have presented intuitive comparisons between the phonon MFP and the structure size^4^. The phonon scattering by boundaries essentially depends on both the surface roughness and the incident phonon wavelength^12^,^13^. This dependence is often characterized by the specularity parameter *p*, which indicates the fraction of phonon energy being specularly reflected by the boundary. For ultra-thin thin films, the confinement of phonons can significantly modify phonon properties such as the dispersion relation^14^. Due to the requirement of forming standing waves^15^, the wavelengths of existing phonon modes must comply with the constraint by the film boundaries^13^. Accordingly, phonon properties such as the density of states (DOS), group velocity and heat capacity, are modified, as has been confirmed by previous molecular dynamics (MD) studies^16^,^17^. Moreover, the change in the phonon dispersions also affects the phonon-phonon interactions, e.g., fewer phonon modes will result in smaller interphonon scattering rates.

Analytical phonon conductivity models based on Boltzmann Transport Equation (BTE), typically in Callaway-Holland form^18^,^19^, have been developed to understand the variation of in-plane thermal conductivity with respect to film thickness through fitting with experimental results^6^,^10^. These models often adopt bulk phonon properties and include the effects of boundary scattering in the relaxation time term. This might be inappropriate for ultra-thin films in which phonon dispersions and scatterings may be different from those in bulk materials. These models also rely on multiple fitting parameters and assume isotropic phonon properties, which might obscure the underlying phonon physics^16^. Meanwhile, the specular parameter *p* for the boundary scattering is often simply assumed to be a constant for all the phonons^20^,^21^. However, this simplification needs further verification. Thin film studies based on MD simulations^16^,^22^ have also been conducted to provide atomic-level information about thermal transport. Classical MD results essentially depend on the quality of interatomic potentials. Considering the fact that few classical potentials including the widely-used Stillinger-Weber^23^ and Tersoff^24^ potentials can accurately reproduce the measured thermal conductivity of bulk Si, it is difficult to directly compare MD results of Si thin films with experimental data. Meanwhile, MD simulation is strictly valid only near or above the Debye temperature while the Debye temperature of silicon (631 K^25^) is much higher than the room temperature.

In contrast, lattice dynamics (LD) calculation targets directly on the transport behavior of individual phonon modes. It is usually combined with BTE to obtain lattice thermal conductivity from kinetics theory^26^. Different from those analytical phonon conductivity models, LD usually adopts full phonon dispersions and considers the mode-wise phonon transport properties, making it more accurate and convenient for spectral analysis, which is of importance for nanoengineering thin films for targeted applications. Lattice dynamics calculation has been successfully used to investigate the relationship between in-plane thermal conductivity and film thickness^16^,^27^. Turney et al.^16^ have implied the importance of considering mode depletion in modeling the thermal transport in thin films. However, few studies have integrated all the aforementioned phonon confinement effects, especially the change in interphonon scatterings, into the prediction of thermal conductivity of thin films. Besides, LD calculation requires the input of phonon dispersion relation and the relaxation time of each phonon mode, which, especially the latter, are often lacking in accurate determination^20^,^28^. In recent years, first-principles calculations have been successfully used to accurately predict the phonon scattering rates and thermal conductivities in simple bulk crystals such as Si and PbTe^29^,^30^. It is very attractive to use the results from first-principles calculations instead of fitted parameters from experiments as the inputs for LD calculations, which may provide more reliable insights into the thermal transport in thin films.

In this study, we have systematically investigated the in-plane thermal transport in Si thin films over a wide thickness range from 80 K to 800 K using the LD method and first-principles calculations. The phonon confinement effects, including the phonon dispersion modification and the change in interphonon scattering, have been integrated into the prediction of thin film thermal conductivity. The relative contributions of these two mechanisms to the thermal transport in ultra-thin films are discussed. The isotope effects in thin films are also explored. The validity of using a constant specularity parameter and bulk phonon properties in thin film studies is further clarified. Thereafter, the effects of the existence of boundary on the spectral phonon transport are discussed.

## Results

### A. Phonon properties and isotope effects

**Figure 1:**
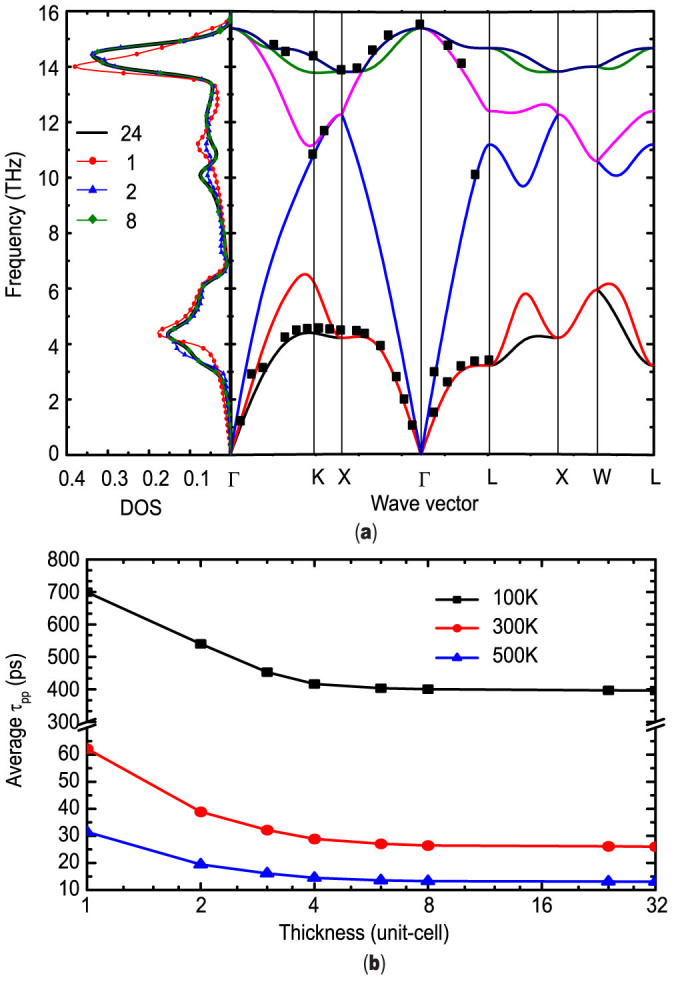
(a) Phonon dispersion relation of bulk silicon along some high-symmetry directions together with the DOS plot for thin films of different thickness (measured in number of unit-cells). The black square dots in the plot of dispersion relation are extracted from the experimental results acquired by G. Nilsson and G. Nelin^31^. (b) Average phonon-phonon scattering relaxation time with respect to the film thickness at different temperatures.

The phonon dispersion relations of bulk Si were calculated based on the harmonic force constants obtained by the first-principles calculations, as shown in Fig. 1(a) together with precedent experimental results^31^. The excellent agreement verifies the accuracy of the harmonic properties of Si from this approach. When the film is attenuated to only a few unit cells thick, the phonon confinement effect limits available phonon modes in the thin film, leading to different harmonic and anharmonic phonon properties from those in bulk Si. Figure 1(a) also shows the DOS variation with respect to film thickness due to this phonon confinement effect. The phonon confinement effects on DOS are shown to vanish when the film thickness increases to 24 unit cells (~13 nm). Similar DOS deviation in very thin films has also been observed from classical MD simulations^16^,^21^. This phonon confinement effect limits available phonon modes in thin film and will also affect the interphonon scattering rate. Figure 1(b) shows the influence of phonon confinement on the average phonon relaxation time limited by the phonon-phonon scattering 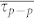 (
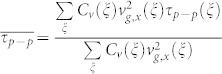
) in thin films of various thicknesses at different temperatures. 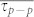 gradually decreases with the increasing film thickness and becomes almost a constant when the thickness is larger than around 24 unit cells (~13 nm).

**Figure 2:**
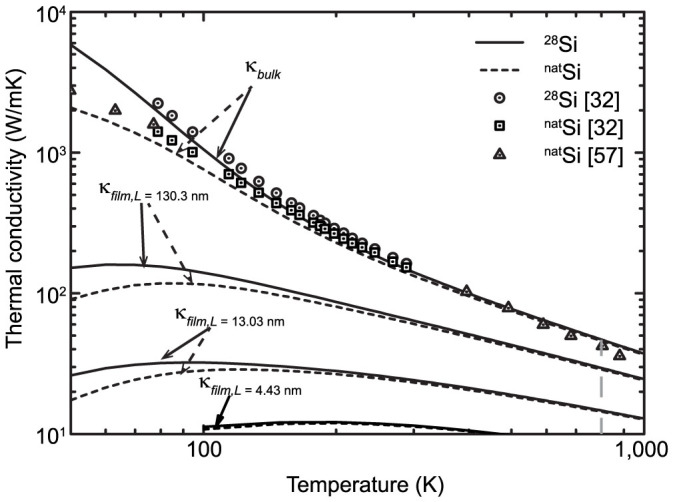
Calculated thermal conductivities of bulk Si and in-plane thermal conductivities of Si thin films with a thickness of 130.3 nm, 13.03 nm and 4.43 nm at different temperatures. The solid lines represent results for isotope-enriched samples and the dashed lines represent the results for the samples with natural isotopic abundance. The experimental results^32^,^56^ for both isotope-enriched^28^Si samples and naturally occurring samples are also shown.

The calculated lattice thermal conductivities of bulk silicon at different temperatures are shown in Fig. 2, together with experimental results. The predicted bulk thermal conductivities of ^28^Si coincide well with the experimental results for ^28^Si in the temperature range from 80 K to 300 K and the predicted bulk thermal conductivities of ^nat^Si are also in good agreement (<15% error) with the corresponding experimental data from 80 K to 800 K, highlighting the accuracy of the current approach. At high temperatures (>800 K), current first-principles calculation tends to overestimate the thermal conductivity because of the ignored higher-order phonon scattering processes, which may play a significant role at elevated temperatures^31^. The calculated thermal conductivity of ^28^Si at 300 K is 141 W/m-K, slightly lower than the experimental result 155 W/m-K^32^, but still close to the result (~145 W/m-K) obtained by Esfarjani using a similar method^33^. This slight underestimation is probably due to the single-mode relaxation time approximation adopted in this work, which may slightly underestimate thermal conductivity, as pointed out by J. Garg et al^34^. A more accurate method to solve BTE is to use the iterative method, as shown by Chernatynskiy et al^35^. However, the discrepancy is small for Si^36^ and is acceptable for thin-film thermal conductivity calculations without fitting with experiments.

Also shown in Fig. 2 are the thermal conductivities of Si thin films with a thickness of 130.3, 13.03, and 4.34 nm. Here the specularity parameter *p* is set to 0. Apparently, the results for thin films are much lower than the bulk values over the entire temperature range. Generally a smaller thickness leads to a lower thermal conductivity but a weaker temperature dependence, because of the relatively stronger boundary scattering. When temperature drops from 300 K to 100 K, the thermal conductivity of bulk ^28^Si increases almost one order of magnitude while the corresponding improvements for the 130.3-nm ^28^Si film and 13.03-nm ^28^Si film are around 170% and 50%, respectively; however, the thermal conductivity of the 4.43-nm film only decreases slightly. The temperature at which the film thermal conductivity reaches its maximum also increases with the decrease in thickness, probably due to the competition among the boundary scattering, interphonon scattering and specific heat variation. For example, the thermal conductivity of the 13.03 nm thick ^28^Si film reaches the maximum at 80 K while that for the 130.3 nm thick ^28^Si film is around 60 K.

The isotope effects are also examined for both bulk silicon and thin films. The thermal conductivity of bulk ^nat^Si with natural isotope abundance (92.2% ^28^Si, 4.7% ^29^Si and 3.1% ^30^Si) is predicted as 133 W/m-K at 300 K, 6% lower than that of pure ^28^Si samples and matching well with the experimental value (7% in Ref. 32). Figure 2 shows that above 200 K the curves for bulk pure ^28^Si and ^nat^Si almost overlap, indicating that the interphonon scattering overwhelms the isotope scattering at high temperatures. Below 200 K, the gap between the bulk thermal conductivities of ^28^Si and ^nat^Si increases with the decreasing temperature, in agreement with the results from Lindsay et al^36^. Although similar phenomena can be found in thin films and the isotopes start to play a role below 200 K, the isotope effects are not significant and become even weaker with the decrease in thickness, implying that the isotope effect cannot match the dominant boundary scattering. For the 4.34 nm film, the isotope effects are almost negligible even at low temperatures. The existence of isotopes will also slightly shift the temperature at which the thermal conductivity reaches the maximum to the right side, e.g., the corresponding temperature for the 13.03 nm film is shifted from 80 K to around 100 K. Overall the isotope effects are not important for thin films at elevated temperatures and they are ignored in the following discussions unless specified otherwise.

### B. Boundary scattering and phonon depletion in thin films

**Figure 3:**
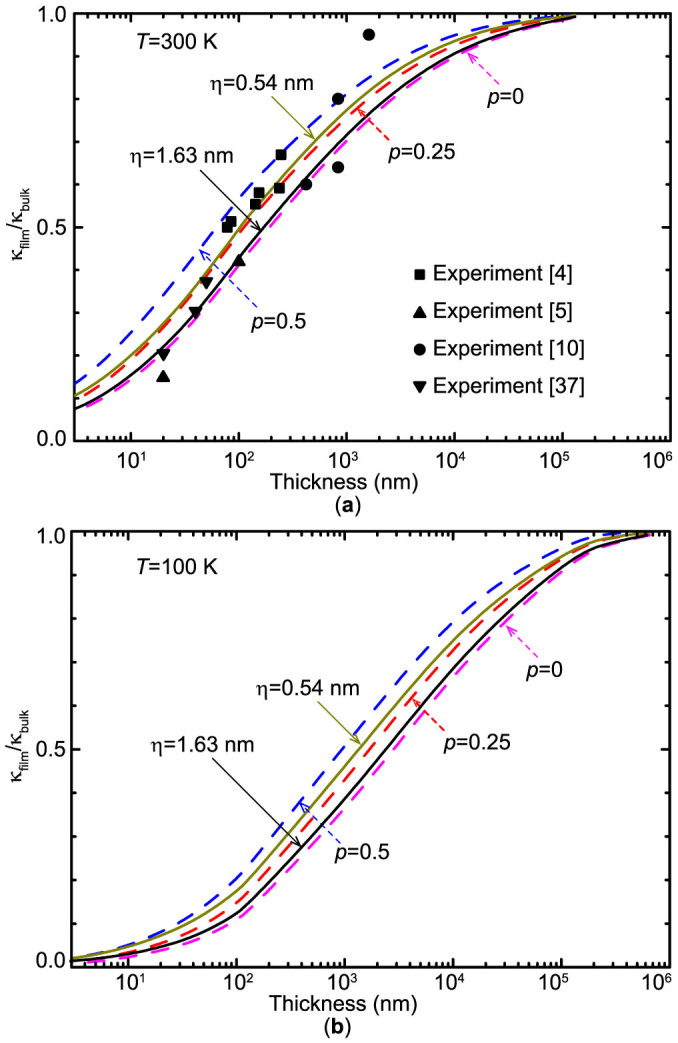
Variation of thermal conductivity of Si thin films with respect to the film thickness at (a) 300 K and (b) 100 K. Different values of the specularity parameter *p*, which are either pre-specified or determined by the surface roughness *η*, are used in the calculation.

As implied in Eq. (8) in the method section, the specularity parameter *p*, which depends on both phonon wavelength and surface roughness, directly affects thermal conductivities of thin films. However, a constant *p* value is often used for convenience in thermal conductivity calculations^20^,^21^. To check the validity of such a gray approximation, by setting *p* as some predetermined constants or a parameter dependent on surface quality and phonon characteristics (Eq. (9)), thermal conductivities of Si thin films with various thicknesses at 300 K are calculated and shown together with experimental results^4^,^5^,^10^,^37^ in Fig. 3(a). Apparently, as observed in experiments, the thermal conductivity of thin film decreases with the attenuation of film thickness. Figure 3(a) also illustrates that at 300 K the effects of boundary scattering may be significant for Si thin films of a thickness below 10 μm (corresponding to 90% of the bulk value). Most experimental results fall into the region between *p* = 0 and *p* = 0.5 curves, implying rather diffusive scattering conditions on the film surface. As the film thickness decreases, the thermal conductivity of thin films is more sensitive to *p*, because the boundary scattering becomes more important for thermal transport. When *p* is determined by the phonon wavelengths and pre-assigned average surface roughness *η*, it is found that the results agree quite well with those obtained by assuming a constant *p* for all the phonon modes. Even though *η* = 0.54 nm approaches a constant *p* = 0.25, a totally diffuse boundary condition (*p* = 0) is still reasonable for most samples since a roughness of only 1 unit cell (0.54 nm) is almost the achievable minimum roughness during fabrication, not to mention the possible existence of a native oxide layer (around 1 nm) on the surfaces of many Si thin films. A similar conclusion has also been drawn from recent experiments^38^ on the spectral transmission in Si thin films. It should be noted that the current model neglects the thickness of native oxide layer and disordered Si surface layer on the Si surface, which may lead to a thermal conductivity slightly higher than the corresponding experimental results. However, the assumption that the specularity can be treated as a constant does not strictly hold at low temperatures, as shown for *T* = 100 K in Fig. 3(b). The *η* = 0.54 nm curve, which almost overlaps with the *p* = 0.25 curve over the entire thickness range at 300 K, deviates from the *p* = 0.25 curve and approaches the *p* = 0.5 curve when the thickness is below 100 nm. This is because the heat-carrying phonons have a longer average wave length at lower temperatures, enabling them less likely to be scattered diffusively at thin film boundary. But for films with a large *η*, e.g., *η* = 1.63 nm, *p* = 0 is still a good approximation at 100 K, indicating a completely diffusive scattering even at low temperatures for surfaces with a large roughness because the dominant phonon wavelength is still not long compared with *η* at this temperature. This agrees well with the recent experimental result^37^. As expected, Fig. 3 shows that the boundary scattering effects will be relatively stronger at low temperatures.

**Figure 4:**
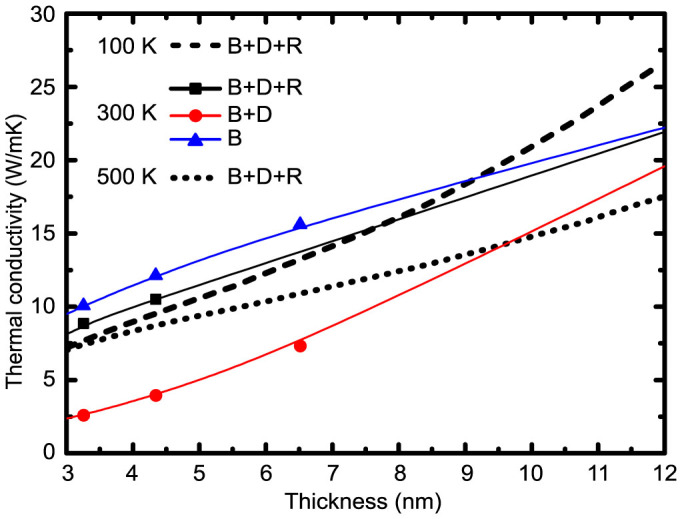
Thermal conductivity in ultra-thin Si films with 1 nm surface roughness at 300 K constrained by different conditions. “B” represents phonon boundary scattering, “D” represents the phonon mode depletion effect and “R” denotes the revision of relaxation time of phonons due to phonon mode depletion in thin films. “D + R” indicates the confinement on phonon, including both the wave length constraint and phonon relaxation time revision.

For ultra-thin films with thickness within the sub-10 nm regime, the boundary scattering, phonon depletion effect and revision of interphonon scattering rate will all contribute to the change of thermal conductivity. However, their individual contribution is yet to be clarified. In Fig. 4, the thermal conductivities of Si thin films with respect to film thickness at 300 K are plotted considering various confinement effects. When the bulk phonon properties (*C_v_*, *v_g_* and *τ_p−p_*) are used and only the boundary scattering is considered, a conventional treatment of boundary effects (denoted as “B”), the thermal conductivity decreases with the reduction of film thickness due to stronger boundary scattering. It is obvious that when the phonon depletion effect due to phonon confinement is also considered (denoted as “B + D”), thermal conductivity can be further reduced because less phonons contribute to thermal transport. The thinner the film is, the fewer phonon modes there exist in the thin film, rendering a larger gap between the “B” and “B + D” curves. However, fewer phonons also result in larger interphonon scattering relaxation times, which can offset the effect of phonon depletion. Considering all these three confinement effects (denoted as “B + D + R”), the calculated thermal conductivities are much higher than the values for the case “B + D”, but they are only slightly lower than those for the case “B” where only the boundary scattering is considered. The small discrepancy between the “B + D + R” line and the “B” line indicates that the phonon depletion effect is largely cancelled by the relaxation time revision. Therefore, the conventionally used “crude” model still works well for ultra-thin films accidentally. Recently Cuffe et al^39^ reported the measurement of the phonon lifetimes of an ultra-thin film as thin as 8 nm. In their analytical fitting part, a simple boundary scattering was added into the phonon-phonon scattering term without considering the phonon depletion effect, which should be important in the sub-10 nm region. However, the fitting results turned to be satisfactory, implying that the phonon mode depletion effect and relaxation time revision effect may counterbalance with each other in such a thin film, which coincides with the current research.

In the frame of “B + D + R”, when temperature is also taken into consideration, some unique behaviors can be observed for ultra-thin films. First of all, for ultra-thin films, the thermal conductivity is almost temperature-independent. For a thin film with thickness of 3 nm, the thermal conductivity changes less than 15% when the temperature rises from 100 K to 500 K, a phenomenon similar to previous MD results^40^. This indicates that the boundary scattering dominates the thermal transport in ultra-thin films in this temperature range. Also shown in Fig. 4 is a crossover between the thermal conductivity variations at 100 K and 300 K. The crossover means that even when silicon has a higher thermal conductivity at 100 K than at 300 K, if the film thickness shrinks to a few nanometers, the situation may be reversed. It is related to the phonon excitation and its interplay with the boundary scattering. For relatively thick films in which the interphonon scattering plays an important role, fewer phonon modes are excited and there are fewer interphonon scattering processes at 100 K than at 300 K, resulting in larger phonon relaxation times or longer MFPs. However, for ultra-thin films, the phonon MFP is essentially determined by the boundary scattering and fewer phonons will be excited to contribute to thermal transport at lower temperatures, resulting in a decrease in the thermal conductivity.

**Figure 5:**
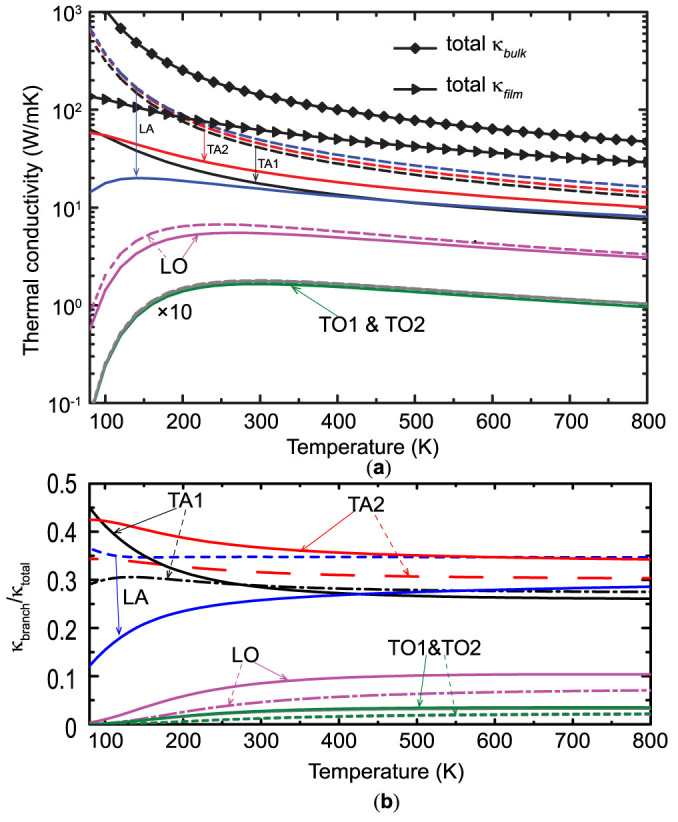
(a) Thermal conductivity contributions from different phonon branches at different temperatures. (b) Relative thermal conductivity contributions from different branches at different temperatures. The dashed lines and solid lines denote bulk Si and the Si thin film, respectively. The thickness of the thin film is 130.3 nm. The values of TO1 and TO2 in (a) have been multiplied by 10. Scattering processes at the boundaries of the thin films are considered as purely diffusive, namely, *p* = 0.

Figure 5(a) shows the thermal conductivity and the contributions from each phonon branch of bulk silicon and a 130.3-nm-thick thin film (denoted with dashed lines and solid lines, respectively) with respect to the temperature. For bulk Si, the thermal conductivity decreases inversely proportional to the temperature due to the increasing Umklapp scattering strength. The optical phonons (including LO, TO1 and TO2) contribute little to the bulk thermal conductivity and their contributions are insensitive to temperature variation above 200 K. At lower temperatures (<200 K), optical phonons hold less contributions while acoustic phonons contributes even more, because less optical phonons are excited below 200 K. The thermal conductivity of bulk Si is dominated by the acoustic phonon branches. Among them, LA modes contribute the most (around 35% at 300 K) while the contributions from TA1 (28% at 300 K) and TA2 (32% at 300 K) are slightly smaller. For the thin film, similar phenomenon is observed, except that the absolute thermal conductivity contributions from acoustic phonons have been suppressed substantially while the contributions from optical phonons are almost intact because of their intrinsic short MFPs limited by the interphonon scattering. The contribution from LA modes also becomes less than those from TA modes below 400 K, indicating LA modes are more sensitive to the boundary scattering due to their relatively longer MFPs. Moreover, at low temperatures, unlike in bulk Si, the absolute LA contribution to the thermal conductivity of thin films does not always increase with the decreasing temperature. Instead, the absolute contribution of LA modes decreases when the temperature drops below 130 K. Meanwhile, the contributions from TA modes still increase with the decrease in temperature. That is because the cut-off frequency of the LA branch is much higher than those of TA branches (shown in Fig. 1(a)) and there are much more high-frequency modes in the LA branch. At low temperatures, the MFPs of these high-frequency acoustic modes are mainly limited by the boundary scattering but their populations decrease significantly with the decreasing temperature, resulting in smaller contributions to thermal transport. Figure 5(b) shows the relative contribution of each phonon branch in bulk Si and the thin film with respect to temperature. For both bulk Si and the thin film, the relative contributions from optical phonons increase with the increasing temperature and start to saturate (around 20% and 10% in total for the thin film and bulk Si, respectively) when the temperature is above 400 K. For bulk Si, the relative contributions of the three acoustic branches vary little with temperature; however, for the thin film, the relative contribution from the LA branch decreases significantly below 400 K and the TA branches dominate at low temperatures.

## Discussion

**Figure 6:**
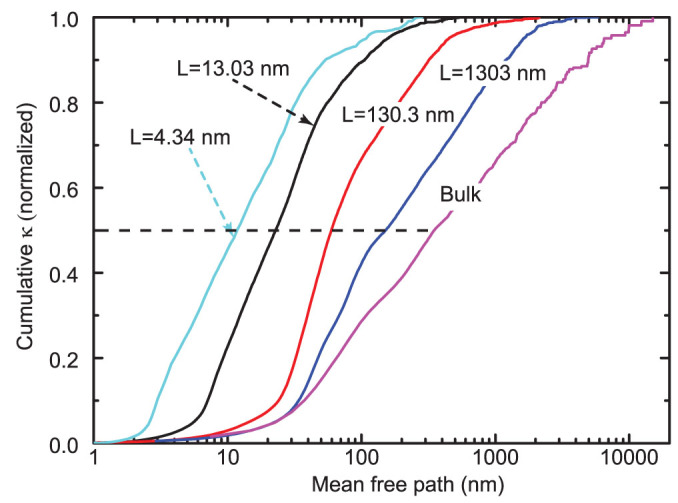
Normalized thermal conductivity accumulation for bulk Si and Si thin films of different thicknesses with respect to phonon mean free path at 300 K.

To understand the effects of the presence of boundary scattering on the phonon MFP and thermal conductivity, by assuming a diffusive boundary condition (*p* = 0), the normalized thermal conductivity accumulations with respect to the phonon MFP for bulk Si and Si thin films of different thicknesses at 300 K have been calculated and are shown in Fig. 6. For bulk Si, although the average phonon MFP in the entire Brillouin zone is calculated to be 47 nm, phonons with a MFP longer than 100 nm contribute around 70% to the thermal conductivity while those with a MFP longer than 1 μm still contribute about 30% to the thermal conductivity. This agrees with the conclusions from previous studies^41^ that a gray approximation may lead to large uncertainties in the analysis. The MFP corresponding to 50% thermal conductivity accumulation for bulk Si is 350 nm, in good agreement with the effective MFP (~300 nm) of dominant phonons derived from experimental conclusion^4^. For Si thin films, as thickness decreases, the relative contributions from phonons with a short MFP increase although the absolute contributions change little while those from long-MFP phonons decrease, leading to the shrinkage in the span of MFP of dominant phonons. Similar results have been reported in a previous work from Monte-Carlo simulations^42^. As shown in Fig. 6, the MFP value corresponding to 50% contribution to thermal conductivity also decreases and becomes more and more comparable to the film thicknesses when the thickness is reduced, indicating stronger boundary scatterings in low-thickness films. Meanwhile, it should be noted that for thin films phonons with a MFP much longer than the film thickness still contribute significantly to the thermal conductivity. This indicates that phonons with a large incident angle contribute more to the in-plane thermal transport.

**Figure 7:**
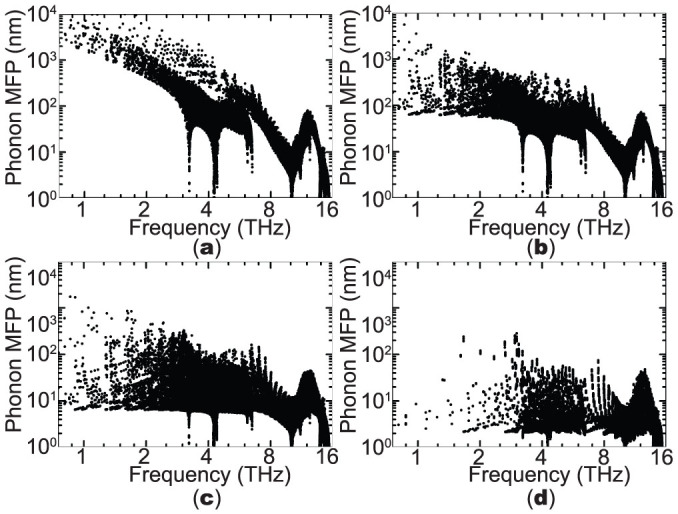
Phonon mean free path distribution with respect to frequency at 300 K for (a) bulk Si and thin films with a thickness of (b) 130.3 nm, (c) 13.03 nm and (d) 4.34 nm.

Figure 7 shows the MFP distribution with respect to the frequency. For bulk Si, the distribution of MFP is significantly uneven and generally phonon MFP decreases rapidly with the increasing frequency. The distribution of MFPs of phonons in bulk Si demonstrates the notable contributions of low-frequency acoustic phonons to thermal transport. The sudden drops in MFP near 4 and 9 THz, corresponding to the cut-off frequencies of the TA and LA branches, are due to the low group velocities at the Brillouin zone boundary. Unsurprisingly, optical phonons generally have short MFPs (<~10 nm). In contrast, in thin films, the MFP distributions become more even. The MFPs of low-frequency acoustic phonons are significantly reduced by the boundary scattering while the MFPs of high-frequency acoustic phonons and optical phonons are much less influenced. However, some low-frequency phonons still remain their original values because their cross-plane group velocities are relatively small, resulting in a smaller scattering rate on the boundary.

**Figure 8:**
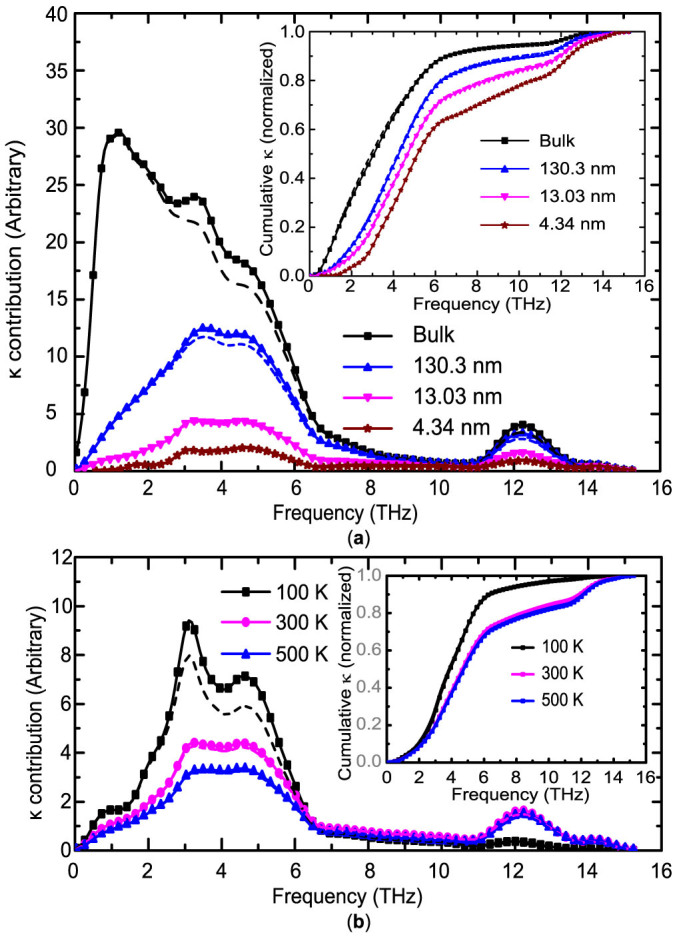
(a) Thermal conductivity contribution with respect to phonon frequency for bulk Si and Si thin films of a thickness of 4.34, 13.03 and 130.3 nm at 300 K. (b) Thermal conductivity contribution of frequency for the 13.03 nm-thick Si films at different temperatures. The solid lines and dashed lines represent the results without and with considering the isotope effects, respectively. The insets show the normalized thermal conductivity accumulations with respect to phonon frequency.

The frequency-dependent contributions to the thermal conductivity for bulk Si and Si thin films with different thicknesses at 300 K are presented in Fig. 8(a). The inset in Fig. 8(a) shows the cumulative thermal conductivity contribution with respect to frequency. Phonons with frequency below 6 THz dominate the thermal transport in bulk Si and these thin films while optical phonons (with frequency from 11 THz to 15.4 THz) contribute only 5% to the thermal conductivity of bulk Si, in line with previous reports^33^. The contributions from low-frequency phonons will be significantly suppressed by the boundary scattering. Both the peak contribution magnitude and position for bulk Si are shifted in thin films while the contributions of high frequency phonons are much less sensitive to the boundaries unless the film thickness is close to their MFP (~10 nm), similar to the less affected participation ratio of optical phonons in porous structures^43^. Figure 8(b) shows the thermal conductivity contribution with respect to phonon frequency in a 13.03-nm Si thin film at different temperatures. The normalized cumulative contributions are shown in the inset. With the decreasing temperature, the contributions from low-frequency phonons (below 6 THz) increase while those from the high-frequency regime (>11 THz) decrease, probably due to the reduction in the interphonon scattering rate and the population of high-frequency phonons. The influence of isotopes on the thermal conductivity contributions is also explored and shown in Fig. 8. Generally, as shown in Fig. 8(a), for both bulk Si and thin films at 300 K, the isotope effects are small and they mainly suppress the contributions from acoustic phonons with a frequency between 3 and 5 THz. When film thickness decreases, the boundary scattering becomes stronger and the isotope effects become relatively weaker. Figure 8(b) also shows that at 100 K, the isotope effect becomes important and it mainly reduces the contributions from acoustic phonons with a frequency between 3 and 5 THz. However, at 300 K and 500 K, the influence of isotopes is negligible, consistent with previous discussions.

In summary, we have systematically investigated the dependence of thermal conductivity of Si thin films on various parameters, including thickness, temperature, isotope effect, and surface roughness. The approach adopted is based on phonon BTE with inputs from the first-principles calculations and lattice dynamics, making it possible to directly compare the predictions with experimental results. The good agreement between the predictions and experimental results indicates the validity of this approach. The phonon confinement effects are also considered in this approach. The investigation of the thermal transport in bulk Si and thin films shows that isotope effects are not important above 200 K. For ultra-thin films with a thickness below ~13 nm, phonon depletion induced by the phonon confinement modifies the phonon spectrum but its effect on thermal conductivity is largely offset by the relaxation time variation. The widely-used gray approximation for the specularity factor is only valid at elevated temperatures or for samples with a large surface roughness. The spectrum analysis shows the dominant phonons for thermal transport in thin films, providing information for tailoring thermal conductivity through nanoengineering.

## Methods

### Phonon thermal conductivity from lattice dynamics

Lattice thermal conductivity can be calculated by solving the phonon Boltzmann Transport Equation (BTE) with inputs from first principles calculations. Despite the higher accuracy of the iterative solution^35^, the single-mode relaxation time approximation is sufficient in determining thermal conductivity of silicon because of the strong non-conserving phonon scattering in the material^36^,^44^ and is therefore adopted here for its easier implementation and its explicit relationship with phonon mean free path. According to the kinetic theory about phonon transport and applying Fourier's law, the lattice thermal conductivity *κ* can be predicted by^34^: 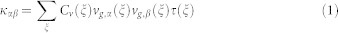
where *κ* is a 2^nd^-order tensor with the subscripts *α* and *β* denoting its components, *C_v_*is the phonon mode heat capacity, **v_g_** = ∂*ω*/∂**q** is the group velocity and *τ* is the phonon relaxation time. This summation is over all phonon modes *ξ*(**q**,*j*) denoted by the wavevector **q** and the dispersion branch *j*.

In Eq. (1), *C_v_*, *v_g_* and *τ* are all mode-dependent. The phonon mode heat capacity *C_v_* is calculated through 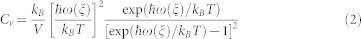
where *ω* is the phonon angular frequency, *k_B_* is the Boltzmann constant, *T* is the temperature and *V* is the volume of the unit cell respectively. Phonon mode heat capacity and group velocity can be determined from the dispersion relations obtained by solving the eigenvalue problem through diagonalizing the Fourier-transformed harmonic interatomic force constants (dynamical matrix)^45^.

Phonon relaxation time *τ* indicates the scattering strength of phonons. In Si crystal, there are generally various scattering mechanisms that limit the phonon transport, including intrinsic phonon-phonon scattering, boundary scattering, and phonon-defect scattering (isotope scattering in this study). Matthiessen's rule is often adopted to sum up the effects of independent multiple scattering mechanisms 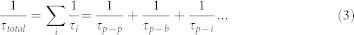
Here *τ_p−p_*, *τ_p−b_*and *τ_p−i_*are the phonon relaxation times limited by the phonon-phonon scattering, boundary scattering, and phonon-isotope scattering, respectively.

### Phonon-phonon scattering

For silicon thermal conductivity calculation, the term of intrinsic phonon-phonon scattering *τ_p−p_* can be predicted by anharmonic LD calculations. Although it is possible to consider the forth-order or even higher order inharmonic effects in LD calculations, which might be important at very high temperatures, three-phonon scattering is the dominant phonon-phonon scattering process below 1000 K. In this work we consider the intrinsic three-phonon scattering process and then the relaxation time *τ_p−p_*is related to the third-order anharmonic interactions^29^

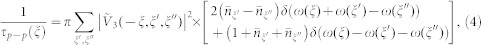
where 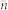 is the equilibrium Bose-Einstein distribution function of phonons. In this process, the conservation of energy and momentum should be satisfied, i.e., *ω*(*ξ*) ± *ω*(*ξ*′) = *ω*(*ξ*″) and **q** ± **q′** = **q″** + **G**, where **G** is either 0 (for normal process) or a reciprocal lattice vector (for Umklapp process). The interaction strength 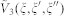 among three phonon eigen modes *ξ*,*ξ*′ and *ξ*″ can be determined by: 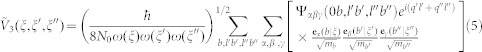
where **e** is the phonon mode eigenvector and Ψ*_αβγ_*(0*b*,/′*b*′,/″*b*″) represents the third-order force constants in terms of atoms *b*, *b′* and *b″* in lattice *0*, *l′* and *l″* of a crystal consisting of *N_0_* cells.

### Isotope scattering

For isotopes, the mass variation model is often adopted with the mass variance given by 
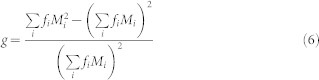
where *f_i_* and *M_i_* are the fraction and mass of the *i^th^* specie of atoms, respectively. According to the model proposed by Tamura^46^, the corresponding relaxation time can be calculated as: 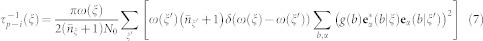
Two different silicon samples, i.e., pure ^28^Si and naturally occurring Si which consists of 92.2% ^28^Si, 4.7% ^29^Si and 3.1% ^30^Si, are considered in this study to illustrate the isotope effects.

### Boundary scattering

Based on the accurately calculated bulk phonon properties, the phonon transport process in thin films can be analyzed accordingly. Assume the coordinate z is along the cross-plane direction, the interactions between phonons and the boundary can be quantified as 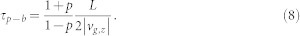
Here 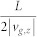 represents the average time duration for a phonon to reach the boundary, provided evenly distributed possibility of emission location and emission angle. The specularity parameter *p*, ranging from 0 to 1, is used to account for the possibility of specular reflection on the surface. It can be either specified as a constant value or determined through the relationship^47^

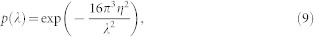
where *λ* is the phonon wavelength and *η* is the average surface roughness.

### Phonon confinement

When the film thickness is comparable to that of wavelength, confinement from the boundaries may affect the thermal transport by changing the wave characteristics of phonon^16^,^48^. This effect is referred to in the current manuscript as “phonon confinement”. It is obliged by the requirement of forming standing waves in the thin film, i.e., phonon wave vector magnitude *q*, which is inversely proportional to the wavelength, should satisfy the constraint 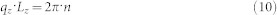
Here *L_z_* is the thickness of thin film and *n* is an integer number. Therefore, in the cross-plane direction, only waves that are accommodated by the thickness of the thin film can exists, leading to a change in phonon dispersion relations and phonon DOS.

The confinement effect applies for an infinite potential well or a periodic boundary condition^48^. Whereas for an ultra-thin film, the surface reconstruction^49^ and the existence of native silicon dioxide layer would defy these assumptions. However, these disorders only affect the first few atomic layers near the surface^50^. For Si films of a thickness larger than 3 nm in this study, the majority of atoms still stay on their lattice sites as in bulk Si. Because of the short-range covalent nature of Si-Si bonds, the interatomic force constants among these inner atoms will not change with the thickness, which has been confirmed in our first-principles calculations. Therefore, the vibration spectra of the main body of the film are close to those of a corresponding film embedded in bulk (see the supplemental materials), but under the constraint of Eq. (10), meaning that the LD calculation is still valid for ultra-thin films. The influence from the surface disorder can be considered in the specularity factor *p*. Then we can obtain the interphonon scattering rate of each mode in thin films according to Eqs. (4) and (5) by only considering the available modes in thin films.

Since the thermal conductivity is obtained by summing up over all the **q**-points in the Brillouin zone, it can be expected from first sight that the phonon depletion will result in a further decrease of thermal conductivity. However, once the phonon confinement effect is taken into consideration, the assumption that phonon relaxation time remains the original value as in the bulk material is no longer valid. Because of fewer phonons available in the Brillouin zone, some of the original scattering channels which satisfy the momentum conservation relation shown in Eq. (4) don't comply with the standing wave requirement and therefore the interphonon scatterings become weaker. Different from previous studies that adopt bulk interphonon relaxation times in the calculation, in this study the relaxation times of thin film phonon modes are recalculated by only considering the three-phonon scatterings among the available modes in thin films.

### Computation details

In this study, a conventional unit cell of silicon was first relaxed within the density-functional theory framework under the Perdew-Burke-Ernzerhof (PBE)^51^ form of the generalized gradient approximation (GGA) for the projector augmented wave method^52^ (PAW) implemented in the Vienna Ab initio simulation package (VASP)^53^. The cut-off energy was set as 500 eV. It yielded a lattice constant *a* = 5.429 Å, perfectly agreeing with the experimental value 5.431 Å^54^. Then a 2 × 2 × 2 supercell was constructed. Thereafter, the so-called direct method^55^ was applied to this supercell to calculate the second and third-order force constants. In this method, atoms were displaced in independent directions. The second-order force constants 
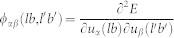
 (*u_α_*(*lb*) is the *α* coordinate of the *b*^th^ atom inside the *l*^th^ unit cell) and the third-order force constants 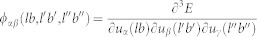 can then be extracted from the energy and force variation. The static first-principles calculations were conducted with a precision as high as 10^−8^ eV for the total energy difference between two self-consistency steps and 5 × 5 × 5 **k**-points (reciprocal space mesh for electronic self-consistent field calculation) to obtain the forces on each atom within the perturbed systems. After the extraction of the harmonic and anharmonic force constants, the frequency and relaxation time of each mode were calculated by conducting a Fourier transformation with a dense **q** mesh (reciprocal space mesh for phonons) scheme 24 × 24 × 24, which has been tested to be enough to yield converged results in the temperature range from 50 K to 1000 K. Further increase in the mesh scale has little effect on the predicted bulk thermal conductivity values.

For thin film calculations only considering phonon depletion effect but no scattering rate revision, we extract the phonon properties, such as phonon frequency and inter phonon scattering relaxation time *τ_p−p_*, from bulk phonon modes, with the consideration of the constraint on phonon wavelength [Eq. (10)]. The group velocities of these available modes in the thin film are also recalculated.

However, for ultra-thin films, both the mode depletion and scattering rate revision effects should be considered. The phonon properties in ultra-thin films, including the group velocities and interphonon scattering relaxation times, cannot be extracted from the bulk phonon properties any more, but rather need to be recalculated. During the calculation, a 24 × 24 × *N* **q** mesh scheme is chosen, where *N* denotes the number of allowable q-points in the cross-plane direction in the thin film. The interphonon scattering relaxation time *τ_p−p_* of each phonon mode in the thin film systems are recalculated according to Eqs. (4) and (5). Different thin film thickness and surface roughness values are specified in order to analyze their effects on the thermal transport in thin films.

## Author Contributions

X.W. and B.H. wrote the manuscript. X.W. prepared all the figures. All authors reviewed the manuscript.

## Supplementary Material

Supplementary Information Supplemental Informaiton
