# Supplementary material for: Computational Study of In-Plane Phonon Transport in Si Thin Films
Source: Sci Rep. 2014 Sep 17;4:6399. doi: 10.1038/srep06399 (PMC4165977; doi:10.1038/srep06399)
Supplement: Supplementary Information — Supplemental Informaiton [file srep06399-s1.doc]

Supplementary Information for

Spectral In-plane Phonon Transport and Isotope Effects in Si Thin Films

Xinjiang Wang and Baoling Huang*

*Department of Mechanical and Aerospace Engineering, The Hong Kong University of Science and Technology, Clear Water Bay, Kowloon, Hong Kong*

* Correspondence and requests for materials should be addressed to B.L.H. (mebhuang@ust.hk,+852-23587181).

In this study, we conducted the structure optimization of Si thin films in vacuum using the first-principles calculations. It is found that, for the relaxed film, the first 2-3 atomic layers (about 0.5 nm) are disordered due to the surface reconstruction. However, the atoms in the inner part of the thin film still remain their lattice sites as those in bulk. Because of the short-range nature of the covalent bonding, the interatomic force constants are also very close to those in bulk Si, as confirmed in the first-principles calculations. Figure S1 compares the phonon density of states (DOS) of the surface layer and that of the inner part within a 3-nm thin film and a significant difference between them can be observed. Therefore, we treat a thin film as a sandwich structure. Since the disordered surface layer is very thin, its contribution to the total thermal conductance is small for films thicker than a few nanometers. To clarify whether the lattice dynamics calculation can still be applied to ultra-thin films, the DOS of the film embedded in bulk with the same thickness as the inner part is also calculated using lattice dynamics with the constraint on phonon wavelength by the boundary. As shown in Figure S1, the DOS of the “embedded” film is very close to that of the inner part, implying that lattice dynamics calculation is still valid for ultra-thin films.


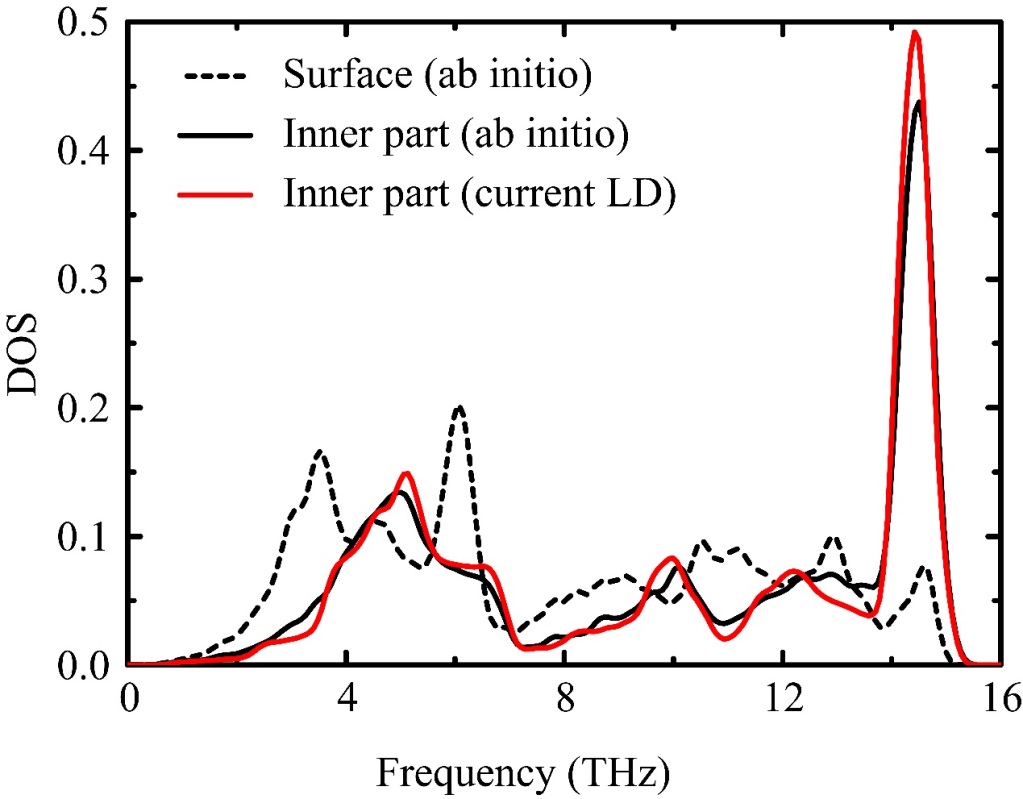


Figure S1. Phonon DOS of the first surface layer and inner part of a relaxed 3-nm Si film from actual “first-principles” lattice dynamics calculation for the film in vacuum. Using lattice dynamics with the consideration of mode depletion, the DOS of the “embedded” thin film of the same thickness as the inner part is also calculated and shown for comparison.
